# NAC and DTT promote TGF-β1 monomer formation: demonstration of competitive binding

**DOI:** 10.1186/1476-9255-3-7

**Published:** 2006-04-11

**Authors:** Frank J Lichtenberger, Christine R Montague, Melissa Hunter, Gwyn Frambach, Clay B Marsh

**Affiliations:** 1 Department of Internal Medicine, Division of Pulmonary and Critical Care, Dorothy M. Davis Heart and Lung Research Institute, The Ohio State University, Columbus, OH, USA

## Abstract

TGF-β plays an important role in the genesis and progression of pulmonary fibrosis. We sought to determine the role of mononuclear phagocytes in the activation of TGF-β and found that freshly isolated peripheral blood monocytes spontaneously released TGF-β. Stimulating these monocytes with GM-CSF or LPS, but not MCSF, augmented the activation of TGF-β. In human monocytes, the free thiol compounds DTT and NAC decreased the activity of TGF-β, without affecting TGF-β mRNA transcription. Both NAC and DTT lessened the biological activity of recombinant active TGF-β in a cell-free system. We found that NAC and DTT reduced dimeric active TGF-β from a 25 kDa protein to 12.5 kDa inactive monomer. This conversion was reversed using the oxidizing agent diamide. Diamide also restored biological activity to NAC or DTT-treated TGF-β. Reduction of TGF-β to monomers could competitively inhibit active dimeric TGF-β and block intracellular signaling events. Our observations suggest that modulation of the oxidative state of TGF-β may be a novel therapeutic approach for patients with pulmonary fibrosis.

## Background

TGF-β is a conserved multi-functional growth factor and is biologically active in femtomolar concentrations [1]. The effects of TGF-β are diverse and include the modulation of cell growth and differentiation, suppression of immune responsiveness, and induction of extracellular matrix production [1]. There are three isoforms of TGF-β (TGF-β1, TGF-β2 and TGF-β3) expressed in humans which are produced by a variety of cells with platelets being a major source. In this study, we will focus on TGF-β1 which will be referred to as TGF-β.

TGF-β is secreted as an inactive latent molecule that is bound non-covalently with its accessory molecule latency associated peptide (LAP)[2]. TGF-β and LAP are derived from the same gene then post-translationally modified. The TGF-β and LAP monomers form homodimers, which join together to form the latent TGF-β heterotetramer (L-TGF-β) or small latent complex. Secreted as L-TGF-β, TGF-β must dissociate from LAP to be biologically active [3], which is achieved *in vivo *by molecules such as thrombospondin-1[4], plasmin [5], and the epithelial cell integrin α_v_β_6_[6]. Once released from LAP, active TGF-β exists as an intensely hydrophobic homodimeric protein composed of two 12.5 kDa monomers linked by a single disulfide bond [7].

TGF-β exerts its biologic impact through binding and activation of type I and type II TGF-β serine/threonine kinase receptors (TGF-βR-I, and TGF-βR-II)[8]. TGF-β is thought to first bind to type II, which then heterodimerizes with type I. Upon crosslinking of the receptor subunits, type II phosphorylates serine and threonine residues located in conserved GS cytoplasmic domain of type I receptor resulting in a conformational change of type I and subsequent activation of signaling pathways [9]. An accessory receptor termed type III, does not signal, but is thought to enhance the association between type I and type II [10]. Ultimately, the signals generated by the TGF-β receptors are relayed to the nucleus by the Smads transcription factors [11].

TGF-β1 is the most prevalent isoform found in human pulmonary fibrotic diseases [12]. In pulmonary fibrosis, collagen-rich connective tissue is inexorably deposited in the lung, interrupting gas exchange. In animal models of pulmonary fibrosis, blocking the binding or function of TGF-β1 with antibodies, [13] or soluble receptors, [14] reduces fibrosis. Furthermore, over-expressing the antagonistic Smad-7 has the same effect as blocking active TGF-β and underscores the role of TGF-β on disease models of fibrosis [15].

The importance of TGF-β1 in fibrotic diseases does not appear to be limited to animal models, as increased levels of biologically active TGF-β1 are found in BAL fluid from patients with fibrotic lung diseases [16]. Moreover, BAL effluents from patients with pulmonary fibrosis demonstrate an overabundance of younger, recently recruited monocyte/macrophages to the lung [17]. These observations suggest a potential relationship between mononuclear phagocytes, TGF-β1 activation, and fibrosis.

It had been previously reported that endothelial cells secrete TGF-β1, and that the cytokine activity is reduced when reacted with thiols [18]. In addition, the free thiol compound N-acetylcysteine (NAC) can modulate the anti-proliferative activity of TGF-β1 [19] and decrease collagen accumulation in the bleomycin-induced mouse model of pulmonary fibrosis [20]. We sought to determine if thiol compounds affected monocyte activation of TGF-β, and if the effect could be measured in a cell free system. We found that human monocytes spontaneously produced TGF-β. The growth factor GM-CSF and LPS each promoted TGF-β activation in human monocytes. NAC and DTT reduced the activity of TGF-β produced by human monocytes, but did not suppress its gene transcription. In a cell-free system, DTT and NAC also blocked the biological activity of purified active TGF-β by creating TGF-β monomers. These monomers competitively inhibited the biological effects of active human TGF-β in vitro as detected by Smad phosphorylation and the TGF-β sensitive luciferase reporter assay. Therefore, modulation of the oxidative state of TGF-β may be a potential therapy for patients with pulmonary fibrosis.

## Methods

### Materials

Human recombinant TGF-β1, purified human active TGF-β1, and recombinant human latent TGF-β1 were purchased from R&D Systems (Minneapolis, MN). Cytokine stock solutions (100 ng/μl) were supplemented with 3% human serum albumin. All culture media, antibiotics, and antifungal agents were obtained from Invitrogen (Carlsbad, CA). Diamide, Ellman's reagent, and DTT were purchased from Sigma (St. Louis, MO).

### Production of TGF-β monomers

TGF-β monomers were prepared by treating 100 μl of TGF-β at 2 ng/μl with 10 μl 1 M DTT, at 37°C for 20 minutes, after which time 10 μl 25% HSA was added to absorb excessive reducing equivalents. Identical vehicle controls were prepared without TGF-β in order to determine that this effect was specific to reduced TGF-β and not an effect of reducing agents coupled with carrier protein.

### Transformed Mink lung epithelial cell TGF-β assay

Mink lung epithelial cells (TMLC cells) transfected with a plasminogen activator inhibitor-1 promoter linked to a luciferase reporter system (kind gift of Dr Daniel Rifkin, NYU) were grown in DMEM high glucose media containing L-glutamine, pyridoxine hydrochloride, and 110 μg/ml sodium pyruvate. and supplemented with 10 U/ml Penicillin G, 10 μg/ml streptomycin G sulfate, 25 ng/ml Amphotericin B, and 10% heat inactivated FBS in a 5% CO_2 _humidified incubator at 37°C. In our laboratory, FBS independently induced luciferase production, therefore trypsinized TMLC cells were washed twice in serum-free media, resuspended to 2.5 × 10^5 ^cells/ml in DMEM without FBS and aliquoted (100 μl/well) in a 96 well plate. The cells were allowed to adhere for 4–6 hours, and then the media was removed and replaced with cell-free supernatant of unstimulated or stimulated monocytes or recombinant protein. Following incubation at 37°C in 5% CO_2 _for 18–20 hours, the cells were then washed twice with PBS and lysed with 50 μl cell lysis buffer (Promega, Madison, WI). The cell lysate (20 μl) was mixed with 90 μL of luciferase assay reagent (Promega) and measured on a Lumat LB 9507(Berthold, Oak Ridge, TN). Luciferase values were normalized to that of media alone in recombinant protein studies. When supernatants of monocytes were used, the value was standardized to that of the media from non-stimulated monocytes.

### TGF-β ELISA

ELISA kit (R&D Systems) was performed according to manufacturer's instructions. Briefly, the capture antibody was diluted to the working concentration in PBS, and was placed on Costar EIA 96-well plates at 100 μl per well. The plate was sealed and incubated at room temperature overnight. The following day each well was aspirated and washed with wash buffer (0.05% Tween 20 in PBS) for a total of five washes using an autowasher then blocked for 1 hour at room temperature with PBS containing 5% v/v Tween 20 and 5% v/v sucrose. Plates were washed, then 100 μl of either isolated protein samples or monocyte supernatants were added per well. Following incubation at 37°C for 1 hour, the plates were washed, then incubated with 100 μl of detection antibody for 1 hour at 37°C and washed. Streptavidin-HRP was added to each well, the plate was covered and protected from light for 20 minutes. The plate was washed as above and 100 μl substrate solution was added to each well and incubated for an additional 20 minutes at room temperature avoiding ambient light. The reactions were stopped with 50 μl of 2 N H_2_SO_4 _and the optical density was measured at 450 nm using MRX plate reader (Dynatech Laboratories) and 570 nm for correction.

### Western blot and immune detection

Akr cells which have detectable phospho-Smad2/3 activity in the presence of TGF-β, were plated on costar 12 well plates at a concentration of 1 × 10^6 ^cells/well in serum-free McCoy's 5A modified media with l-glutamine. Three hours after treatment the cells were lysed with triple detergent lysis buffer (50 mM Tris-HCl pH 8.0, 0.15 M NaCl, 1% Triton X-100, 0.1% SDS, 5 mg/ml Sodium deoxycholate, NaF 1 mM, and Na_3_VO_4_1 mM) and protein concentration quantitated using the BioRad Assay (Hercules, CA). Samples prepared from either Akr whole cells lysates or from recombinant protein were separated on polyacrylamide SDS gels, under non-reducing conditions, (15% for TGF-β, 10% for Phospho-Smad 2) and transferred to nitrocellulose membrane. Nitrocellulose membranes were then incubated with either rabbit anti TGF-β (sc-146, Santa Cruz Biotechnologies), rabbit anti-phospho-Smad2 (cat# 06-829, Upstate Biotechnologies, Lake Placid, NY), or mouse anti Smad2/3 (cat# S66220, Transduction Laboratories, Louisville KY.) antibodies at a dilution of 1:1500 followed by incubation with the appropriate secondary antibody and then detection using ECL (Amersham, Arlington Heights, IL). For equal loading considerations, the membrane used for the anti-phospho-Smad2 blot was reblotted for Smad2/3.

### Reclamation of TGF-β activity with diamide

TGF-β (10 μg) was incubated with either 10 μl of 100 mM NAC or 5 μl of 100 mM DTT for 15 minutes at room temperature, then 10 μl of 100 mM diamide was added and samples were incubated for an additional hour. The samples were diluted with 400 μl PBS containing 1% HSA as a carrier protein and dialyzed on a 10,000 MWCO Slide-A-Lyser for 1 hour to remove diamide, which was shown to have adverse effects on the reporter cells. The dialyzed sample was then used in the TMLC assay.

## Results

### NAC and DTT reduce TGF-β production and activation by human monocytes but do not reduce TGF-β mRNA expression

**Figure 1:**
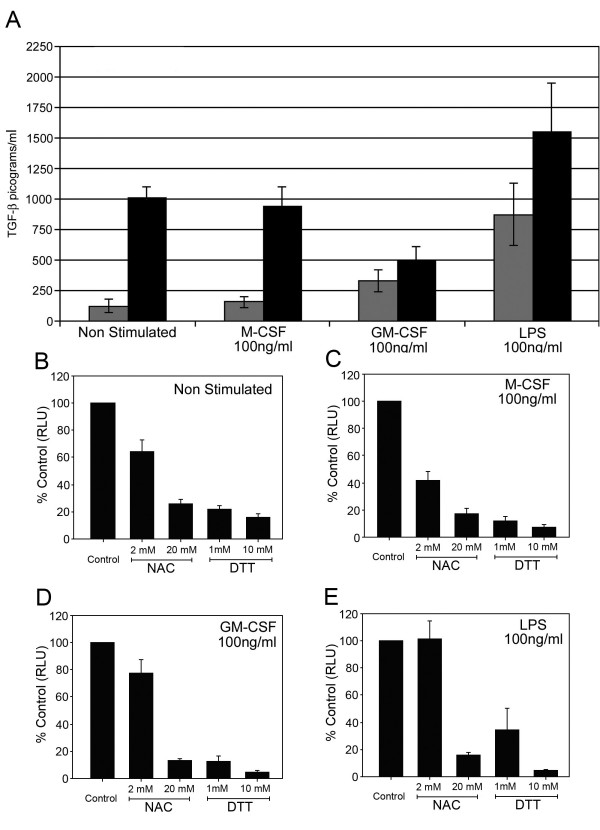
**TGF-β activity produced by monocytes is decreased by NAC and DTT**. Human monocytes (5 × 10^6^/condition) were incubated in media alone or with M-CSF (100 ng/ml), GM-CSF (100 ng/ml) or LPS (100 ng/ml) for 18 hours. (A) Cell-free supernatants were harvested and analyzed by TMLC assay for active TGF-β (*Grey bars*) and compared against total TGF-β produced (*Black bars*). M-CSF, GM-CSF and LPS induce more active TGF-β from monocytes than that induced by incubation with media alone, p = 0.06 for M-CSF, p < 0.05 for GM-CSF and p < 0.01 for LPS. These data are composite of six independent studies. These same conditions are shown titrated against increasing concentrations of NAC and DTT, non stimulated (**B**) or incubated with M-CSF (**C**), GM-CSF (**D**) or LPS (**E**). After incubation, cell-free supernatants were evaluated for TGF-β by TMLC assay and were expressed as percent control of samples treated without NAC and DTT. NAC (20 mM) and DTT (both 1 and 10 mM) reduced TGF-β activation and production by all the stimulating agents and from cells left not stimulated (p < 0.01 versus samples not treated with NAC or DTT). The data shown are representative of four measurements each from six independent studies.

Since TGF-β can bind receptors on human macrophages and monocytes in lung tissue [22] and we predict that TGF-β has a potential role in mediating the development of pulmonary fibrosis, we sought to understand how TGF-β activity was regulated by monouclear cells. To measure active TGF-β, Rifkin, et al. developed a mink lung epithelial cell (TMLC) that has been stably transformed with a plasminogen activator inhibitor-1 promoter (a common Smad activated gene) linked to a luciferase reporter system. The binding of active TGF-β to TGF-βRI and TGF-βRII on these cells induces the activation of Smad 2/3, resulting in luciferase production. In contrast, latent TGF-β does not induce luciferase production in this reporter system, limiting detection to the active TGF-β component, therefore we used the TLMC assay to access the activity of TGF-β produced by human monocytes. Monocytes were obtained from the Red Cross from healthy volunteers following informed consent and, purified as previously described [24]. As shown in Figure 1A, monocytes spontaneously released latent TGF-β (*black bars*). GM-CSF and LPS, but not M-CSF induced the activation of TGF-β by human monocytes (p < 0.05) (Figure 1A, *gray bars*). Although LPS induces monocytes to activate TGF-β [25], it has not been reported that M-CSF or GM-CSF act similarly. Of these stimuli, LPS resulted in the largest release of active TGF-β by these monocytes.

We next examined the effect of either NAC or DTT on the activation of TGF-β by monocytes. Both NAC and DTT reduced the activity of TGF-β from untreated monocytes (Figure 1B) or monocytes treated with either M-CSF (Figure 1C), GM-CSF(Figure 1D), or LPS (Figure 1E).

**Figure 2:**
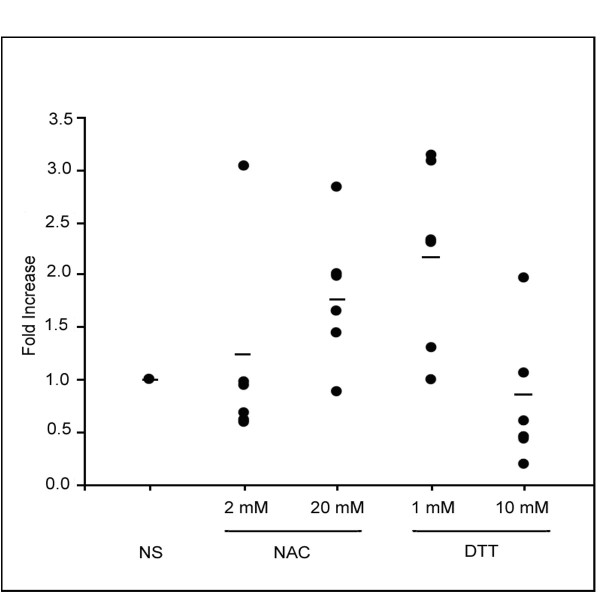
**NAC and DTT do not consistently reduce TGF-β mRNA synthesis from human monocytes**. Monocytes (10 × 10^6 ^per condition) were treated with 2 mM NAC, 20 mM NAC, 1 mM DTT, or 10 mM DTT and incubated for 24 hours. Data points represent fold induction over non-stimulated; mean is represented by horizontal bar. Only DTT 10 mM reduced TGF-β mRNA versus non-stimulated control cells (p < 0.05) other conditions were not significantly different statistically. This data represents three independent studies.

We then determined if the reduction of TGF-β production by NAC and DTT in monocytes was due to inhibition of gene transcription. The treated monocytes were then subjected to real time PCR analysis to examine TGF-β mRNA levels. As shown in Figure 2 the addition of NAC or low concentrations of DTT had no effect on the TGF-β mRNA levels. However, TGF-β mRNA expression was slightly decreased only by the addition of the 10 mM DTT (Figure 2).

### NAC and DTT block recognition of purified active TGF-β

**Figure 3:**
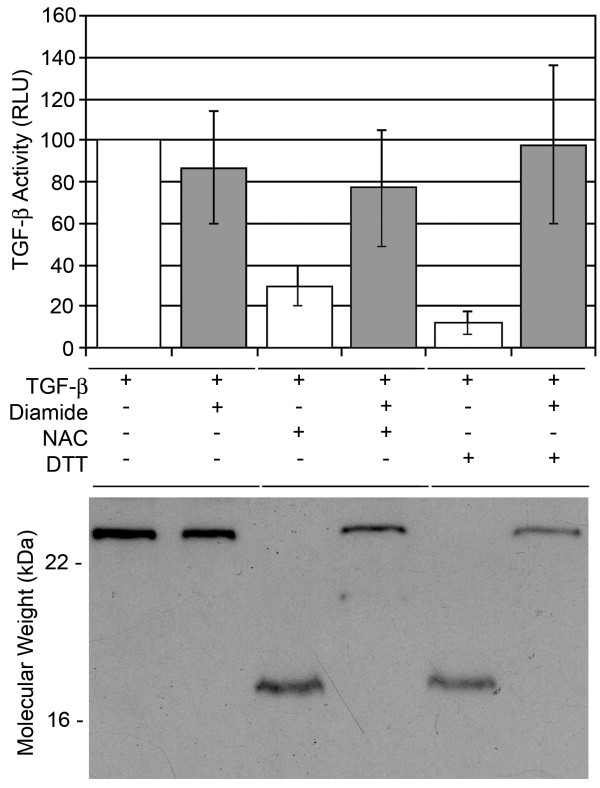
**Activity of purified TGF-β NAC is dependent on Redox state**. Purified active TGF-β (R&D Systems) was treated with either NAC or DTT as described, was then incubated in the absence or presence of 10 mM diamide for two hours at 37°C. Samples were dialyzed for two hours then either (A) incubated with TMLC cells to measured luciferase production or (B) separated by polyacrylamide gel electrophoresis and subjected to western blot analysis TGF-β. Data represents percent of control ± standard error where control is TGF-β sample untreated (p < 0.05 for NAC 2 mM and 10 mM versus TGF-β alone). Diamide restored activity levels to that of TGF-β alone (p > 0.05 of TGF-β alone versus TGF-β + NAC (20 mM) or TGF-β + DTT (10 mM) + diamide). Recombinant human TGF-β migrated as a ~25 kD protein that was reduced to a ~12.5 kD protein by the addition of NAC or DTT. Data shown in (B) is a representative of six independent experiments.

To study the relationship between the redox state of TGF-β and its activity we incubated recombinant TGF-β with either NAC or DTT. The treated TGF-β was dialyzed against PBS to remove the NAC and DTT trace then assayed for activity using the TMLC assay. We found that only 20 mM NAC whereas both DTT concentrations (10 mM and 1 mM) were able to reduced TGF-β activity measured by TMLC luciferase assay (Figure 3A) (p < 0.001 for active TGF-β1 incubated with NAC (20 mM) and DTT (10 mM) versus active TGF-β1 (1 ng/ml) alone). We further confirmed our observations by incubating the reduced TGF-β with the oxidizing agent diamide. We found that diamide treatment restored the detection of TGF-β from NAC and DTT-treated samples (Figure 3A).

We hypothesized that NAC or DTT reduced the biological activity of purified TGF-β by reducing TGF-β homodimers to monomers. We therefore analyzed the generation of monomers from the active human (25 kDa) homodimers with either NAC or DTT in the absence or presence of diamide. We detected TGF-β monomers with the apparent molecular weight of 12.5 kDa in samples treated alone with either NAC or DTT by Western blot analysis (Figure 3B) suggesting that these reducing agents promoted the formation of TGF-β monomers. Notably, addition of diamide restored detection of the 25 kD isoform of TGF-β.

### TGF-β1 monomers reduce biological effects of active TGF-β1

**Figure 4:**
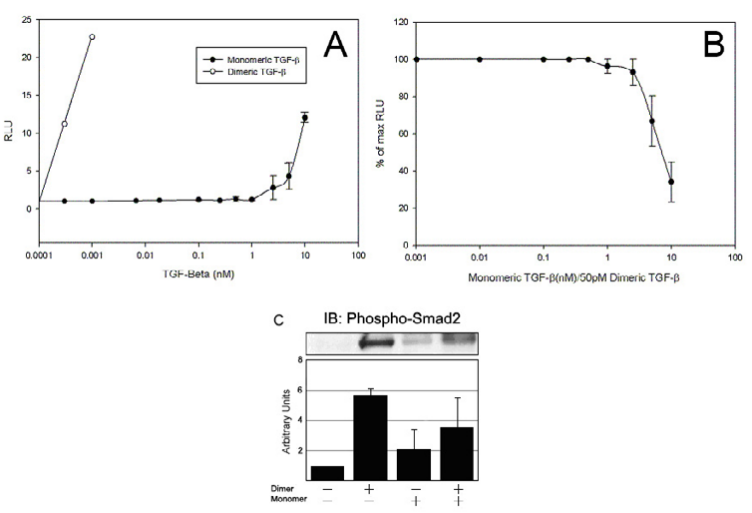
**TGF-β treated with DTT functions as competitive inhibitors**. (**A**) Comparison of the luciferase activity released by TMLC cells that were incubated with recombinant human TGF-β (°) or DTT-treated TGF-β (•). Recombinant TGF-β is activate below 25 pM, while the DTT-treated TGF-β only showed activity at 5 nM. (**B**) TMLC cells (1.0 × 104 TMLC cells/well) were incubated with recombinant human TGF-β at 50 pM and increasing concentrations of DTT-treated TGF-β. Competitive agonist effect of DTT-treated TGF-β was apparent at ~100 fold higher concentration. The data shown are representative of three independent studies done in quadruplicate. (**C**) Mouse epithelial Akr cells (1.0 × 10^6 ^cells/condition) containing TGF-β receptors, were treated with 5 ng/ml of rhTGF-β and phosphorylated SMAD2/3 was measured by western blotting. The blots were quantitated by densitometry (*bar graph*) and 50 ng/ml monomeric TGF-β lowers the impact of the dimeric TGF-β (p < 0.01 vs non stimulated, p = 0.127 vs dimer treated). The data are representative of eleven independent studies. Error bars represent the standard error of the mean.

Since we could generate monomeric forms of TGF-β, which failed to be recognized by our reporter assay system, we were interested to understand the biological function of this protein. We therefore hypothesized that TGF-β monomers would act as competitive inhibitors of the cellular activation induced by TGF-β homodimers. TGF-β monomers were unable to induce luciferase production in the TMLC assay at concentrations below 1 nM (Figure 4A). Notably higher concentrations exceeding 1 nM retained some agonist activity measured by TMLC assay. Compared to dimeric TGF-β which was capable of eliciting an effect at concentrations below 0.001 nM, the level of activation by the TGF-β monomers was 1000-fold less active (Figure 4A). To examine whether the monomeric TGF-β form could act as a competitive inhibitor, increasing concentrations of monomeric TGF-β was incubated with the homodimeric isoform. As predicted, TGF-β monomers generated by treating active TGF-β with DTT, reduced luciferase production in TMLC cells when used in 50-fold excess to active TGF-β (Figure 4B).

We further confirmed that the inhibitory effect of the monomeric TGF-β was modulated within the cell. Since Smad2 is the downstream activator of TGF-β stimulation, we examined whether the monomeric TGF-β form could prevent the activation of Smad2. As shown in Figure 4C, the addition of monomeric TGF-β alone to Akr epithelial cells failed to activate Smad2 when compared to cells treated with the homodimeric form. Notably, incubation with both the dimer and 50-fold excess of monomer TGF-β resulted in a reduction of Smad 2/3 activation by monomeric TGF-β to dimeric TGF-β (Figure 4C). Of note, DTT-treated monomers were extensively washed, and quenched with Human Serum Albumin prior to using in competitive inhibitor studies to reduce the likelihood that inhibitory activity reflected residual DTT left in the culture.

## Discussion

Latent TGF-β is primarily regulated by post-translational activation of TGF-β from LAP [3]. However, the mechanism of TGF-β activation in human cells is incompletely understood. Interestingly, immunohistochemical analysis of pathological tissue from humans with pulmonary fibrosis shows that TGF-β localizes to areas with increased amounts of macrophages in the lung [22,23]. To define the mechanism used by these cells to activate TGF-β, we found that the addition of GM-CSF or LPS, but not M-CSF to these monocytes induced the activation of TGF-β. Interestingly, NAC and DTT significantly lessened TGF-β produced by these monocytes, irrespective of whether growth factors or LPS was added. To investigate the mechanism involved, we initially found that NAC or DTT did not consistently decrease mRNA expression of TGF-β, suggesting that the level of regulation was post-transcriptional. Subsequently, we realized that NAC and DTT also reduced the activity of recombinant human TGF-β in a cell-free system and hypothesized that these agents lessened TGF-β activity by reducing disulfide bonds of the active homodimers.

We next found that treatment of active TGF-β with NAC or DTT reduced the size of detectable purified TGF-β from 25 kD to 12.5 kD. Diamide restored detection of the 25 kD isoform of TGF-β in the presence of NAC and DTT and also restored the biological activity of TGF-β. Next, we wondered if these 12.5 kD TGF-β monomers would competitively inhibit cellular activation induced by active TGF-β homodimers. If used in 50-fold molar excess, TGF-β monomers inhibited cellular activation by active TGF-β homodimers assayed by either a luciferase-based reporter system or by assessing Smad 2 activation. Thus, TGF-β monomers may have potential as biological modifiers of the biological effects of active TGF-β.

The production and activation of TGF-β plays an important role in regulating the acute and chronic phases of tissue injury. TGF-β is a growth factor that plays an important role in the regulation of the inflammatory response [24], and in fact, transgenic animals lacking TGF-β die of overwhelming inflammation in utero or at young ages[25,26]. In addition to anti-inflammatory properties, TGF-β promotes fibrosis as part of altered tissue repair [27,28]. For example, in pulmonary fibrosis, TGF-β is found in the lung and the elevation in levels of TGF-β correlate with the extent of fibrosis. In animal models of pulmonary fibrosis, administering antibodies to TGF-β or blocking signal transduction modulated by active TGF-β by over-expressing the inhibitory Smad-7 blocks fibrosis [13-15]. Thus, defining the specific mechanisms regulating the production and activation of TGF-β may have therapeutic opportunities to help patients with fibrotic diseases.

As opposed to recent trends using molecular targeted therapy for cancer, most treatments for patients with inflammatory or fibrotic diseases have not used similar strategies. Currently, well-defined molecular or cellular targets are lacking in patients with fibrosis, although TGF-β has been found to play a causal role. Our data suggests that agents like NAC and DTT decrease the biological function of TGF-β and liberate TGF-β monomers. Modifying active TGF-β homodimers to inactive monomers results in loss of biological activity, and the generation of a competitive TGF-β receptor agonist. This new therapeutic opportunity links to other strategies that interfere with the production and activation of TGF-β including decorin [28], antiplasmin [29] or other cytokines like interferon-γ [30].

## Conclusion

Based on our results, NAC and DTT reduce the biological activity of TGF-β after activation. Since activation of TGF-β is thought to be the most powerful regulator of the biological activity of TGF-β, reducing the biological activity of TGF-β after activation heightens the therapeutic opportunity to treat patients with tissue fibrosis. These data also provide a tentative mechanism for reducing agents like NAC as treatment for pulmonary fibrosis [32-34]. One potential mechanism for the effect of NAC in these studies may be reduction in the biological activity of TGF-β, reducing fibrosis. In this paper, we suggest that TGF-β monomers resulting from treatment with NAC and DTT may compete with TGF-β dimers for TGF-β receptors. Thus, altering the reduction-oxidation state of the environment may influence the function of TGF-β. We believe that understanding the molecular regulation of TGF-β activation and recognition may provide opportunity to intercede on this process.

## Abbreviations used

TGF-β – Transforming Growth Factor-beta, NAC – N-Acetylcysteine, DTT – Dithiothreitol, PBS Phosphate buffered Saline, RPMI – Roswell Park Memorial Institute, DMEM – Dulbecco's Modified Eagle Medium, DTNB – Ellman's Reagent. LAP – Latency-Associated Peptide.

## Competing interests

The author(s) declare that they have no competing interests.

## Authors' contributions

FL designed and carried out all experiments expect for the PCR experiments. He drafted and reviewed the final manuscript. CM designed and carried out the PCR experiments. MH critically reviewed the manuscript. GF assisted in data collecting and in performing the experiments and additionally assisted in the design and implementation of the monocytes and performed statistical analysis on the data. CM coordinated the experiments and participated in drafting the manuscript.

## Supplementary Material

Additional File 1 Animation clip showing the active form of TGF-β, a dimer, binding to it's receptor RII, then crosslinking with RI to initiate signaling. Click here for file

Additional File 2 Animation clip showing reducing agents creating TGF-β monomers which interfere with signaling. Click here for file

Additional File 3 **TGF-β mRNA quantikine kit to quantify TGF-β1 mRNA produced my monocytes treated with growth factors and reducing ageants**. Figure Legend: NAC does not affect TGF-β mRNA synthesis from human monocytes treated with Growth Factors. Monocytes (5 × 10^6^/condition) were treated with M-CSF(100 ng/ml), GM-CSF(100 ng/ml), and LPS (100 ng/ml) in the presence or absence of 20 mM NAC. TGF-β was measured using specific mRNA quantikine kit. **Method**: 5 × 10^6 ^Monocytes per condition, treated with M-CSF, GM-CSF, or LPS(all 100 ng/ml.) In the presence or absence of 20 mM NAC. **Results**: No statistaical difference between in mRNA production, between any condition. Click here for file

Additional File 4 **Ellman's Reagent to quantify Thiol reduction by NAC, DTT, and oxidation by Diamide**. **Figure Legend: DTT and NAC increase detection by Ellman's reagent**. DTT (0.01, 0.05, 0.1, 0.5, 1.0, 5, 10 mM) and NAC (0.01, 0.05, 0.1, 0.5, 1.0, 5, 10 mM) were added to DTNB. The lowest concentration of NAC that was detectable to reduce the disulfide bond in DTNB was 2.0 × 10^-9 ^M, well below what was used in our experiments with TGF-β. To these concentrations, increasing amounts of Diamide (0.1, 0.2, 1.0, 2.0, and 10 mM) were added and the ability to reoxidize DTNB was noted as absorbance at 410 nm. 10 mM diamide was not able to reoxidize DTNB at the highest concentrations of DTT, but was able to reoxidize 10 mM NAC treated DTNB. These data represent four redundant measurements from 3 independent studies. The line for 1.0 was left off the graph for simplicity of viewing. **Method**: Ellman's reagent (3,3'-dithio-bis(6-nitrobenzoic acid), DTNB) at 1 mmol in PBS with 10 mM EDTA was placed in a 96 well plate at 100 μl per well. Identical plates were made for measuring the reducing power of NAC and DTT under physiological conditions. Concentrations of 0, 0.01, 0.05, 0.1, 0.5, 1.0, 5, and 10 mM NAC and DTT were titrated against 0, 0.1, 0.2, 1.0, 2.0, and 10 mM diamide. The absorbance was checked on an EL_x _808 at 410 nm using 570 nm as a correction for the absorbance of the plates. **Results**: **NAC and DTT reduce disulfide bonds which is reversed by diamide**. To confirm that the concentrations of NAC and DTT we used in these studies were sufficient to reduce disulfide bonds, we used Ellman's reagent to detect formation of free sulfhydryl residues. Treatment of Ellman's reagent with NAC or DTT induced a 0.98 ± 0.02 and 1.6 ± 0.1 molar ratio increase in free sulfhydryl groups, respectively. Diamide was able to re-oxidize the disulfide bond at equimolar concentrations of the reducing agents, to form a colorless compound. Complete oxidation was noted at 10-fold excess of diamide to NAC and all but the highest concentration of DTT. Click here for file
