# Supplementary figures and images for: NAC and DTT promote TGF-β1 monomer formation: demonstration of competitive binding
Source: J Inflamm (Lond). 2006 Apr 11;3:7. doi: 10.1186/1476-9255-3-7 (PMC1475835; doi:10.1186/1476-9255-3-7)

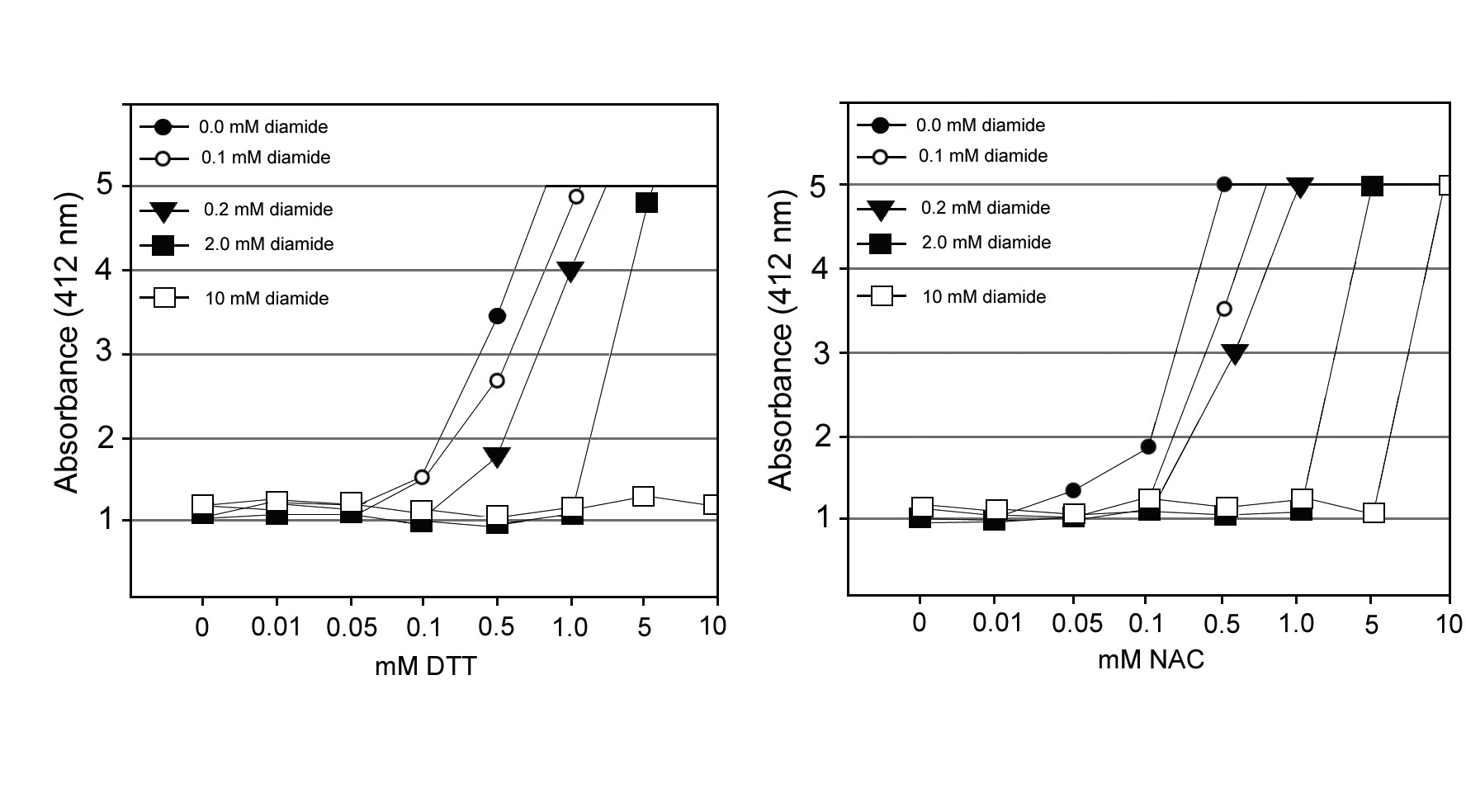

Supplement: Additional File 1 — Animation clip showing the active form of TGF-β, a dimer, binding to it's receptor RII, then crosslinking with RI to initiate signaling. [file 1476-9255-3-7-S1.jpeg]

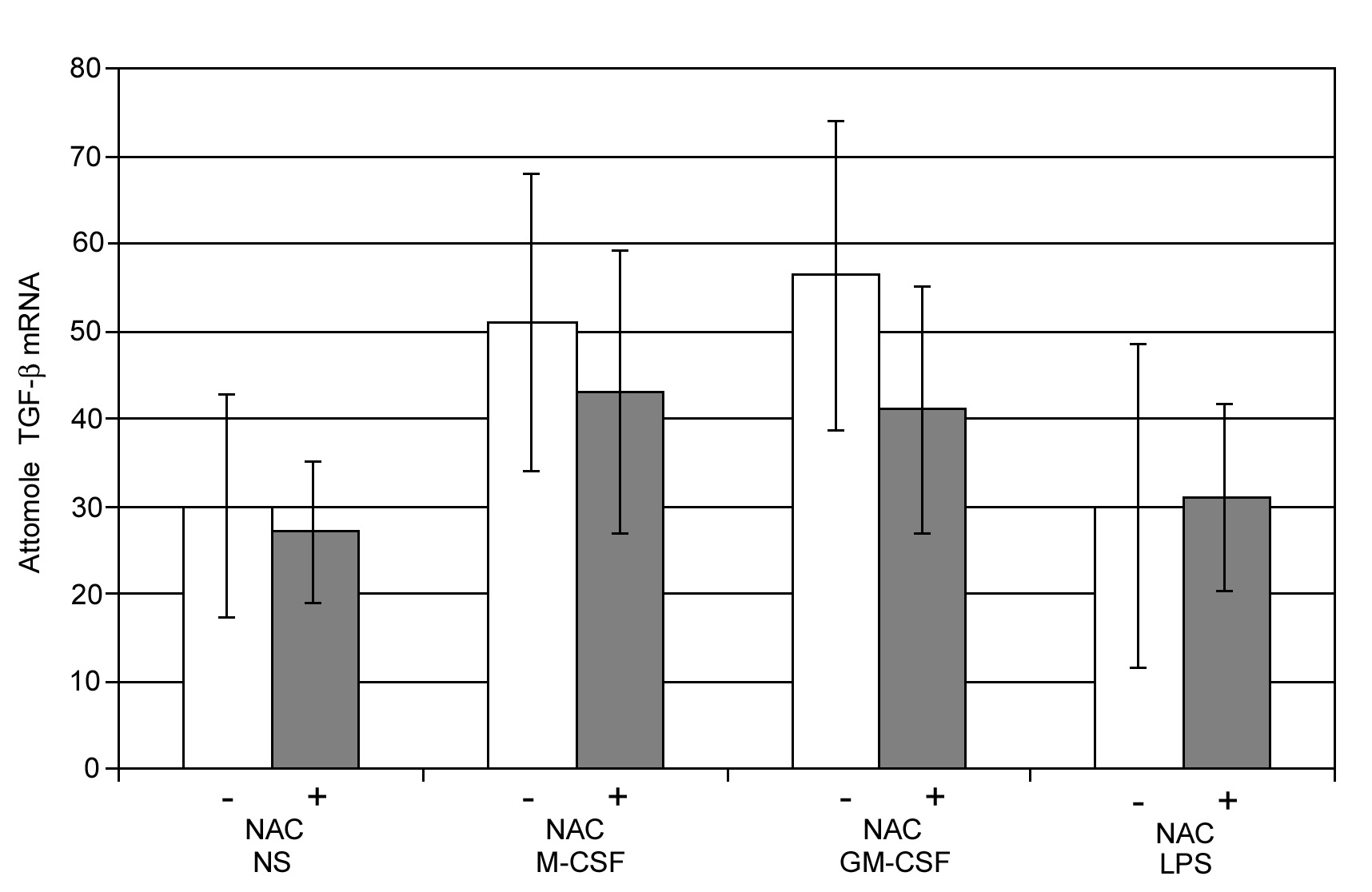

Supplement: Additional File 2 — Animation clip showing reducing agents creating TGF-β monomers which interfere with signaling. [file 1476-9255-3-7-S2.jpeg]
